# Magnetoelectric PVDF–Cobalt Ferrite Films: Magnetostrictive and Magnetorotational Effects, Synergy, and Counteraction

**DOI:** 10.3390/nano15070487

**Published:** 2025-03-25

**Authors:** Oleg V. Stolbov, Yuriy L. Raikher

**Affiliations:** Laboratory of Dynamics of Disperse Media, Institute of Continuous Media Mechanics, Russian Academy of Sciences, Ural Branch, 614018 Perm, Russia; sov@icmm.ru

**Keywords:** magnetoelectric composite film, magnetostrictive particles, PVDF, cobalt ferrite particles, combined striction/rotation effect

## Abstract

Numerical modeling of the direct magnetoelectric (ME) effect in a PVDF–cobalt ferrite (CFO) composite film has been performed. The problem is solved within the framework of the mesoscopic RVE approach, where each elementary cell contains three particles with varying mutual positions. Both modes of mechanical stress generation are taken into account: magnetostrictive and magnetorotational, i.e., changes in the shape and rotation of the particle as a whole. Depending on the sign of the magnetostriction constants, these sources of piezopolarization can either enhance or reduce the overall ME effect. A significant dependence of the ME effect on the mutual arrangement of CFO particles in the cell has been discovered; for instance, the effect is minimal when the particles are closest to each other. In other words, clustering is a negative factor. In a system where the magnetic moments of the magnetically hard CFO particles are ordered, the maximum ME effect is attained when the poling direction is at an angle of about $40^{\circ }$ to the film plane. As it turns out, a fairly good estimate of this angle can be obtained from the solution of a single-particle problem; the main contribution here comes from the ‘diagonal’ components of the piezotensor: $d_{31}$ and $d_{33}$. The ‘tangential’ component $d_{15}$ plays a special role: changing its sign can reverse the polarity of the charge generated on the film.

## 1. Introduction

Magnetoelectric (ME) transducers have numerous applications as sensors and actuators [1,2], energy harvesters [3,4], and tissue-regeneration activators [5,6,7,8,9], etc. In terms of efficiency, composite systems by far surpass single-phase multiferroics, and because of that, the architecture of such systems always includes mechanical stress as the transfer link between the magnetic phase and the piezoelectric phase (direct ME effect) or the piezoelectric phase and the magnetic phase (inverse ME effect).

### 1.1. Magnetostrictive Composites

In conventional practice, the trigger for the direct ME effect is magnetostriction—the change in the shape of a ferromagnetic object (particle) upon magnetization. As a result, a ferroparticle embedded in a composite material becomes a source of mechanical stress, causing piezopolarization in the surrounding medium. This applies equally to solid two-component systems such as ferromagnet (FM) + piezoelectric (PE) ceramic and to two- and three-component polymer composites: FM + PE polymer or FM + PE polymer + solid PE. For this reason, powders of materials with high magnetostriction constants, primarily cobalt ferrite ${\mathrm{Co}}_{1-x}$${\mathrm{Fe}}_{2+x}$$O_{4}$ (CFO) and Terfenol-D, are used as the magnetic phase in ME composites.

Presently, the most appropriate and widely available polymer piezoelectrics are PVDF (polyvinylidene fluoride) and its copolymers. These compounds have a complex supramolecular structure, where only the crystalline β-phase exhibits strong piezoelectric response [10,11,12]. It is the state of this phase that determines the efficiency of the ME composite; therefore, to create a favorable orientational ordering of the piezoelectric domains, PVDF films are subjected to poling (treatment with a strong electric field) or mechanical processing (uniaxial or biaxial stretching).

In the course of the synthesis of composite films, CFO nanoparticles are added to the uncured PVDF. See, for example, [13,14,15,16]. The polar nature of PVDF molecules ensures good adhesion of the polymer to the ferrite particles, so the latter can be considered firmly ‘glued’ into the matrix.

The *magnetostrictive* mode of the ME composite response is excited upon magnetization. Under the action of the field H, the filler particles change their shape and become centers of mechanical stresses. These stresses either directly affect the surrounding PE phase (two-component ME composite) or disturb the polymer matrix, which, in turn, transmits the forces to the PE particles (three-component ME composite).

In multidomain ferromagnetic particles, to ensure a magnetostrictive response, a certain bias field $H_{0}$ must be applied to the sample. By that, the operating point of the ME sensor or transducer is set. In response to the applied test field $H_{t}$, such a composite generates an electric signal: a potential difference Δφ appears on the film. For $H_{t}\ll H_{0}$, the electrical response is linear in $H_{t}$, so the efficiency of ME conversion in the film is usually characterized by a certain ‘specific susceptibility’: coefficient $\alpha =\Delta \varphi /(l\cdot H_{t})$, where *l* is the film thickness. In the case where the directions of the magnetic moments of the filler particles are aligned—which is the case considered below—no bias field is required.

### 1.2. Magnetoactive (Magnetorotational) Composites

A particle of magnetically hard ferrite, such as CFO, possesses its own permanent magnetic moment μ, so that any field H not collinear with μ creates a torque that tends to rotate the particle as a whole to the position μ‖H. For a particle in a composite, this action obviously leads to the appearance of intrinsic mechanical stresses. Note that these stresses, however, are not related to the magnetostrictive effect, as they are not associated with a change in the shape of the particles. We term this way of ME signal excitation *magnetoactive* (by analogy with magnetoactive elastomers) or *magnetorotational*, since the mechanical state of the matrix is disturbed by the rotation of each particle as a whole without changing its shape. Thus, in a magnetorotational composite, the cause of the ME effect is the same as in a magnetostrictive one, but the source of the induced stress is different. From general considerations, it follows that any ME composite should exhibit both effects: magnetostrictive and magnetorotational. However, in the literature on both research and technology of composite polymer magnetoelectrics, the attention is exclusively focused on the magnetostrictive mechanism; see, for example, reviews [4,17,18,19,20,21,22]. This should not be surprising: after all, in experiments, it is quite difficult to separate the contributions of the magnetostrictive and magnetorotational effects.

## 2. Model of a Polymer Composite ME Film

### 2.1. Problem Statement

The goal of this work is to analyze, by means of numerical modeling—with explicit consideration of interparticle magnetic and elastic interactions—the relative contributions of the magnetostrictive and magnetorotational mechanisms to the overall ME effect in a composite film. In Ref. [23], such a comparison was performed within the RVE (representative volume element) approach for the simplest case: a film where each representative cell contains a single particle. Although such a model, like any RVE scheme, to some extent takes into account the mentioned interparticle interactions, this consideration is significantly incomplete, as it is carried out just via the imposition of cyclic boundary conditions at the cell borders.

This limitation is overcome below by constructing an RVE scheme as an assembly of representative cells, each of which contains three particles with varying mutual positions. As it turns out, the ME effect in such a film notably depends on the configuration of the particle triad: it increases with their mutual separation and decreases with their approach.

Another modification in the formulation of the problem under consideration is as follows. In the work [23], for simplicity of analysis, the particles of some reference ferrite with positive uniaxial magnetic anisotropy and the same magnetostriction were considered. The calculation presented below takes into account that in composites of the PVDF@CFO type, ferrite particles possess, as they should for CFO, magnetic anisotropy, and magnetostriction of the cubic type.

**Figure 1:**
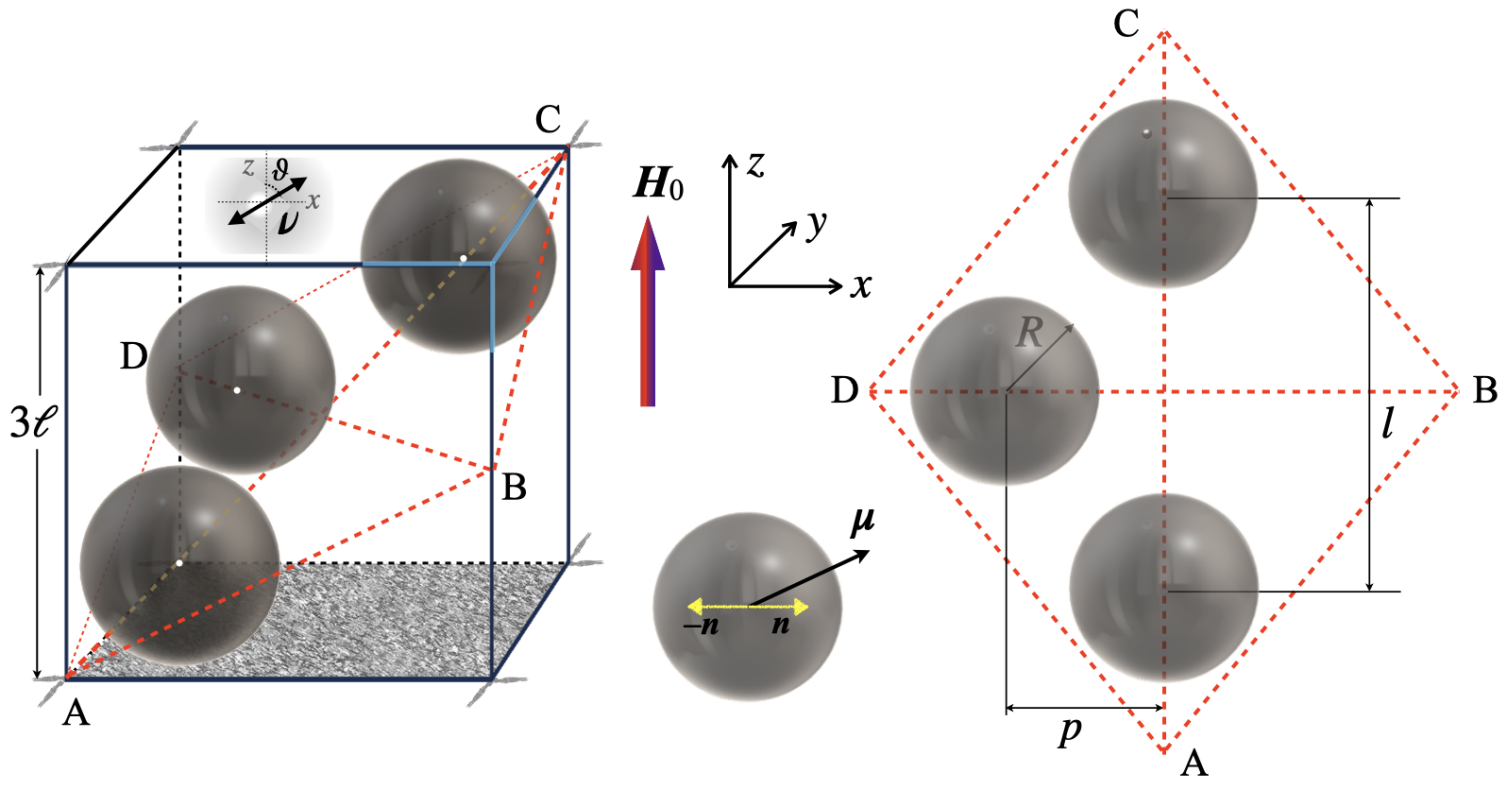
Schematic representation of a cubic RVE cell containing three particles in the diagonal plane of the cube; the angle ϑ defines the poling direction ν; the coordinate system used is shown.

The internal organization of the considered RVE cell is schematically shown in Figure 1. The cell has the shape of a cube with an edge of 3ℓ, inside which there are three spherical magnetically hard particles with equal radii $R=(1/\sqrt[{3}]{3})\ell \approx 0.69\ell$ and identically directed axes of cubic anisotropy. Given this particle size, the volume concentration of ferrite in the cell, and hence in the model film, is 4π/81≈0.155. The film is assembled from such elementary cells arranged in an infinite layer whose lower surface is fixed on a solid plane.

The centers of all particles lie in the diagonal plane of the cube; this rhombus is shown separately in Figure 1. One of the particles is fixed inside the obtuse angle of the rhombus and touches the faces of the cube; the distance from its center to the axis of the rhombus is p=1.23ℓ; the other two particles are located symmetrically on the larger diagonal of the rhombus, their centers separated by a distance *l*, which varies from the maximum approach (1.63ℓ) to the maximum separation (2.78ℓ).

The residual volume of the cell is filled with a continuous medium (matrix), to which the material properties of PVDF are ascribed. The matrix is assumed to have undergone the poling procedure, which turned it into a single piezoelectric domain, the direction of the anisotropy axis of which is characterized by unit vector ν.

### 2.2. Magnetostrictive Deformation of a CFO Particle

We assume that the FM particles are single-domain but large enough so that at the given temperature, they do not transit to a superparamagnetic state. This means that the particle is uniformly magnetized, and its magnetic state is characterized by a single vector—the magnetic moment μ=MVm; here *M* is the magnetization of the ferrite, *V* is the particle volume, and m is a unit vector. The action of the field on such a particle is described by the Stoner–Wohlfarth model [24,25].

The magnetic anisotropy of CFO particles is cubic and positive [26]. In such a ferrite, there are three pairs of easy magnetization axes, their directions coinciding with the edges of the cube in the coordinate frame, whose axes are parallel to the crystallographic lattice vectors. Thus, to describe the anisotropy, one may introduce three mutually orthogonal unit vectors: $n^{\left(1\ldots 3\right)}$, whereas vectors $-n^{\left(1\ldots 3\right)}$ also indicate easy magnetization directions.

Application of magnetic field H causes magnetostriction (change in shape) of the particle. Transforming the general definition of cubic magnetostriction [27] to the notation adopted here, the strain $\lambda_{q}$ in the direction q (here q is the unit vector) experienced by a single-domain particle with magnetization M=Mm can be presented in the form

(1) $$\begin{array}{c} \lambda =\frac{3}{2}\lambda_{100}\left[m_{x}^{2}q_{x}^{2}+m_{y}^{2}q_{y}^{2}+m_{z}^{2}q_{z}^{2}\right]+3\lambda_{111}\left(m_{x}m_{y}q_{x}q_{y}+m_{x}m_{z}q_{x}q_{z}+m_{y}m_{z}q_{y}q_{z}\right), \end{array}$$

where the magnetostriction constants are taken from experiments; for CFO, $\lambda_{100}$ is negative, and $\lambda_{111}$ is positive [28,29]. The strain tensor for the considered case has the form

(2) $$e_{ik}=\frac{3}{2}\left(\begin{array}{ccc} \lambda_{100}m_{x}^{2} & \lambda_{111}m_{x}m_{y} & \lambda_{111}m_{x}m_{z}\\ \lambda_{111}m_{x}m_{y} & \lambda_{100}m_{y}^{2} & \lambda_{111}m_{y}m_{z}\\ \lambda_{111}m_{x}m_{z} & \lambda_{111}m_{y}m_{z} & \lambda_{100}m_{z}^{2} \end{array}\right);$$

it is easy to verify that contraction $e_{ik}q_{i}q_{k}$ yields exactly expression (1).

Let in the initial state (no external field and no applied stresses) the magnetization be directed along the Ox axis. Then, according to (2), the magnetostrictive strain tensor $e_{ik}^{\left(0\right)}$ has a single non-zero component: $e_{xx}^{\left(0\right)}=\frac{3}{2}\lambda_{100}$. This value should be ascribed to spontaneous magnetostriction—the deformation that occurs when the ferrite is cooled below the Curie point. Since spontaneous magnetostriction does not depend on the applied field, only the difference $e_{ik}^{\left(m.\mathrm{strict}\right)}=e_{ik}-e_{ik}^{\left(0\right)}$ is of interest for the sought-for solution.

Further on, the linear approximation is used, i.e., we assume that the applied field H induces only small perturbations in the magnetization of the particles: $m=(1,\delta m_{y},\delta m_{z})$, so that $|\delta m_{y}|,|\delta m_{z}|\ll 1$; for a magnetically hard ferrite in a field of moderate magnitude, such an approximation is quite justified. Then Equation (2) admits linearization and gives

(3) $$e_{ik}^{\left(m.\mathrm{strict}\right)}=\frac{3}{2}\lambda_{111}\left(\begin{array}{ccc} 0 & \delta m_{y} & \delta m_{z}\\ \delta m_{y} & 0 & 0\\ \delta m_{z} & 0 & 0 \end{array}\right).$$

From this it follows that the elongation along the direction q is

(4) $$\lambda_{q}=e_{ik}^{\left(m.\mathrm{strict}\right)}q_{i}q_{k}=3\lambda_{111}\left(q_{x}q_{y}\delta m_{y}+q_{x}q_{z}\delta m_{z}\right).$$

To illustrate the properties of the magnetostrictive response of cubic symmetry, let us assume that the particle is under the action of a field directed along the Oz axis, and this creates only the perturbation $\delta m_{z}$. According to Formula (4), such a change in magnetization causes deformation of the particle neither in the perturbation direction q=(0,0,1) nor across it—along q=(1,0,0). The occurring magnetostrictive strain is non-zero only in the ‘oblique’ direction, and this response is maximal near the angle of $45^{\circ }$ ($q_{x}=q_{z}$) between the directions of the equilibrium magnetization and its perturbation.

### 2.3. Magnetic Torque Acting on a Particle

Simultaneously with the magnetostrictive component, the magnetorotational component of the ME effect also arises. Indeed, at H=0 and in the absence of other external stimuli, the magnetic moment μ of the particle dwells parallel to one of the available anisotropy axes: $\mu \|\pm n^{\left(\alpha \right)}$. A single-domain particle is assumed to be uniformly magnetized almost to saturation, so its magnetic susceptibility in the direction of the axis $n^{\left(\alpha \right)}$ is negligibly small. This allows us to represent the susceptibility tensor of the particle as

(5) $$\chi_{ik}=\chi \left(\delta_{ik}-n_{i}^{\left(\alpha \right)}n_{k}^{\left(\alpha \right)}\right),$$

where χ is the characteristic value of susceptibility determined by the particular magnetization model.

We limit ourselves to the case of not-too-strong external fields, i.e., the range within which the magnetic moment does not switch between alternative easy axes. Thus, the chosen initial direction $n^{\left(\alpha \right)}=n$ is the only ‘easy axis’ of the problem; this allows us to simplify the notation by omitting the index (α). In the Stoner–Wohlfarth model, the above limit on the field magnitude is established by the relation $H<K/M_{s}$, i.e., we consider the range of fields smaller than the minimum coercive force; here *K* is the cubic anisotropy constant, and $M_{s}$ is the saturation magnetization of the ferrite. Under these conditions, the particle magnetizes linearly as

(6) $$M=M_{s}n-\frac{M_{s}}{H_{A}}\left(n\times \left(n\times H\right)\right),$$

where $H_{A}=2K⁄M_{s}$ is the reference anisotropy field. Then, the torque acting on the magnetic moment on the part of the magnetic field takes the form

(7) $$L_{\mu }=\left(\mu \times H\right)=M_{s}V\left(m\times H\right)\left[1-\frac{1}{H_{A}}\left(n\times H\right)\right].$$

Under quasi-static conditions, $L_{\mu }$ is equal to the torque $L_{n}$ exerted on the particle anisotropy axis that tends to rotate it to the position n‖μ. Since the particle is ‘glued’ into the composite, this rotation is hindered by the torque due to elastic mechanical stresses in the deformed matrix. In the considered case, these torques are opposite and equal in their magnitudes. According to a standard rule [30], vector $L_{\mu }$ transforms into magnetic contribution to the stress tensor. For the configuration n‖Ox and H‖Oz, this tensor near the particle surface has the form

(8) $$\sigma_{ik}^{\left(m.\mathrm{rot}\right)}=\frac{1}{2}M_{s}H\left(\begin{array}{ccc} 0 & 0 & 1\\ 0 & 0 & 0\\ 1 & 0 & 0 \end{array}\right),$$

and its intensity decreases with the distance from it.

Let us compare the tensor (8) with the stress tensor due to the cubic magnetostriction of the particle at the same mutual orientation of n and H. Expressing the magnetization perturbations in (3) with the aid of Formula (6), one obtains for the magnetostrictive strain

(9) $$e_{ik}^{\left(m.\mathrm{strict}\right)}=\frac{3}{2}\lambda_{111}\frac{H}{H_{A}}\left(\begin{array}{ccc} 0 & 0 & 1\\ 0 & 0 & 0\\ 1 & 0 & 0 \end{array}\right),$$

The magnetostriction of a particle ‘glued’ into the matrix creates a perturbing strain in the latter and, as a consequence, elastic stresses. Assuming that PVDF obeys Hooke’s law at small strains, the stress state of the matrix in the vicinity of the particle can be expressed through tensor (9) as

(10) $$\sigma_{ik}^{\left(m.\mathrm{strict}\right)}=2G_{p}e_{ik}^{\left(m.\mathrm{strict}\right)}=3G_{p}\lambda_{111}\frac{H}{H_{A}}\left(\begin{array}{ccc} 0 & 0 & 1\\ 0 & 0 & 0\\ 1 & 0 & 0 \end{array}\right),$$

where $G_{p}$ is the shear modulus of the matrix. As can be seen, the symmetric off-diagonal tensors $\sigma_{ik}^{\left(m.\mathrm{strict}\right)}$ and $\sigma_{ik}^{\left(m.\mathrm{rot}\right)}$ have identical structures. This means that in the configuration n‖H, the magnetostrictive and magnetorotational effects affect the piezoelectric matrix similarly. Obviously, in the case of $\lambda_{111}>0$, both tensors have the same sign, and the magnetostrictive and rotational stresses add up; if the signs are different $\lambda_{111}<0$, the effects counteract. The appendix shows how the polarization of PVDF, induced by stresses in the form (8) or (10), depends on the direction of the piezoelectric anisotropy axis.

## 3. Energy Functional of the Composite

### 3.1. Energy of the Composite with a Piezoelectric Matrix

The description of the model system is formalized as follows. The elementary RVE cell is considered to be the composition of several spaces: three regions $\Omega_{m,\beta }$, occupied by magnetic particles β=1,2,3, and the volume $\Omega_{p}$ filled with the polymer. The cell as a whole is embedded in the computational domain—the ‘empty’ space Ω whose boundaries in ±Oz directions are remote from the cell proper by a distance much greater than its thickness 3ℓ.

Below, all calculations are performed under the assumption of small strains since in a matrix like PVDF, mechanical stresses induced by fields up to 3 kOe, which act on the considered ferrite particles, cannot cause significant rotations or switchings. In this approximation, the strain tensor is expressed through the components of the displacement vector u in a standard way:

(11) $$e=\frac{1}{2}\left(\nabla u+\nabla u^{\;T}\right),\;\delta e=\frac{1}{2}\left(\nabla \delta u+\nabla \delta u^{\;T}\right),$$

where the superscript denotes matrix transposition. Then, for the mechanical stress tensors in the corresponding regions of the cell, one has

(12) $$\sigma_{m,\beta }=\sigma_{\Omega_{m,\beta }}=2G_{m}\left[e-e^{\left(m.\mathrm{strict}\right)}\right]+\frac{G_{m}\nu_{m}}{1-2\nu_{m}}\mathrm{Tr}\left(e-e^{\left(m.\mathrm{strict}\right)}\right)I,$$

(13) $$\sigma_{p}=\sigma_{\Omega_{p,\beta }}=2G_{p}\left[e-e^{\left(\mathrm{piezo}\right)}\right]+\frac{G_{p}\nu_{p}}{1-2\nu_{p}}\mathrm{Tr}\left(e-e^{\left(\mathrm{piezo}\right)}\right)I,$$

where $G_{p}$ and $G_{m}$ are the shear moduli of the matrix and ferrite, $\nu_{p}$ and $\nu_{m}$ are their Poisson’s ratios, and I is the unit tensor. In Formula (12), $e^{\left(m.\mathrm{strict}\right)}$ is the magnetostrictive strain of the ferrite particle, and in Formula (13), $e^{\left(\mathrm{piezo}\right)}$ is the piezoelectric strain of the matrix; as is known [31], neither of them generates mechanical stresses in the medium where it arises.

According to the basic theory [31], the part of the thermodynamic potential density of the composite associated with the piezoelectric effect in the matrix, i.e., with the appearance of an electric field E in it, is

(14) $$\Phi =-\frac{1}{2}\sigma_{p}\;\cdot \;\cdot C^{-1}\;\cdot \;\cdot \sigma_{p}-\frac{1}{8\pi }\;E\;\cdot \;\left(\varepsilon_{p}-I\right)\;\cdot \;E-E\;\cdot \;P_{0}-E\cdot \gamma \;\cdot \;\cdot \sigma_{p},$$

here, $\varepsilon_{p}$ is the dielectric permeability tensor of the matrix, and $P_{0}$ is the spontaneous polarization, i.e., a parameter independent of the applied stress. In the linear response approximation, stresses are related to strain through a set of elastic constants given by the 4th-rank tensor C. In Formula (14), the 3rd-rank tensor γ (piezotensor) characterizes the piezoelectric properties of the polymer; it is symmetric in the last pair of indices [31].

The variation of the potential Φ gives

(15) $$\begin{array}{c} \delta \Phi =-\;\;\left[P_{0}+\frac{1}{4\pi }\left(\varepsilon_{p}-I\right)\;\cdot \;E+\gamma \;\cdot \;\cdot \sigma_{p}\right]\;\cdot \delta E-\left(C^{-1}\;\cdot \;\cdot \sigma_{p}+E\;\cdot \gamma \right)\;\cdot \;\cdot \delta \sigma_{p}=\\ -P\cdot \delta E-e\cdot \cdot \delta \sigma_{p}. \end{array}$$

From that formula, follow the expressions for the total electric polarization vector and the strain tensor of the matrix:

(16) $$P=P_{0}+\frac{1}{4\pi }\;\left(\varepsilon_{p}-I\right)\;\cdot \;E+\gamma \;\cdot \;\cdot \delta \sigma_{p},\;e=C^{-1}\;\cdot \cdot \sigma_{p}+E\;\cdot \gamma .$$

Inverting the second of Equation (16), one obtains

(17) $$\sigma_{p}=C\cdot \cdot \left(e-E\cdot \gamma \right)$$

that allows us to obtain an explicit expression for the piezoelectric strain tensor:

(18) $$e^{\left(\mathrm{piezo}\right)}=E\cdot \gamma .$$

### 3.2. Derivation of the Variational Equation

For further calculations, it is convenient to switch to thermodynamic potential Ψ=Φ+σ··e, defined in the variables E and e. After the corresponding Legendre transformation, variation of Ψ takes the form

(19) $$\delta \Psi =-P\cdot \delta E-\sigma_{p}\;\cdot \;\cdot \delta e.$$

Substituting here $\sigma_{p}$ in the form (17) and keeping only the terms linear in γ, one arrives at the expression

(20) $$\delta \Psi =-\;\;\left[P_{0}+\frac{1}{4\pi }\left(\varepsilon_{p}-I\right)\;\cdot \;E+\gamma \;\cdot \;\cdot e\right]\;\cdot \delta E+\left[C\;\cdot \;\cdot \left(e-E\gamma \right)\right]\;\cdot \;\cdot e.$$

In the considered model, it is assumed that the piezopolymer matrix (PVDF) is in a single-domain state, i.e., it possesses uniaxial piezoelectric anisotropy, the direction of which is characterized by the unit vector ν. (Do not confuse the components of the vector ν with the notations of the Poisson’s ratios $\nu_{p}$ and $\nu_{m}$). This state is imposed by appropriate poling and/or mechanical processing [10,11,12]. In this case, piezotensor γ can be represented in the form

(21) $$\gamma_{i,kl}=A\nu_{i}\delta_{kl}+B\nu_{i}\nu_{k}\nu_{l}+\frac{1}{2}C\left(\nu_{l}\delta_{ik}+\nu_{k}\delta_{il}\right),$$

so that the expression for the piezopolarization is

(22) $$P^{\left(\mathrm{piezo}\right)}=\gamma \cdot \;\cdot \sigma_{p}=A\sigma_{p}\cdot \nu +B\mathrm{Tr}\left(\sigma_{p}\right)\nu +\frac{1}{2}C\left(\nu \cdot \sigma_{p}\cdot \nu \right).$$

In the physics of piezoelectrics, a special system of notations is used for the coefficients relating electrical and mechanical quantities; see, for example, [32]. In particular, the electric polarization created by elastic stresses is presented as

(23) $$P_{i}=d_{ik}t_{k},$$

where the matrix of material parameters d has dimensions 6 × 3, and the six-dimensional vector t is composed of the components of $\sigma_{p}$ taking into account that the stress tensor is symmetric.

For PVDF and polymers with a similar structure, matrix d has three independent components, viz. $d_{33}$, $d_{31}$ and $d_{15}$ [12,33]. Comparing notations (23) with the standard form of this equality, $P_{i}=\gamma_{i,kl}\sigma_{kl}$, allows one to express the components of tensor γ through the components of matrix d, which gives

(24) $$A=d_{31},\;B=d_{33}-d_{31}-d_{15},\;C=d_{15}.$$

We note the importance of these equalities: without them, no quantitative calculations can be performed since the pertinent books and handbooks describe piezoelectrics exclusively in terms of coefficients $d_{ik}$.

Consider now variation δU of the total energy of the RVE cell. To do this, we integrate the terms of the variation δΨ in accordance with their definitions over the domain Ω and its components $\Omega_{p}$ and $\Omega_{m}$. We also include in this expression the variations (i) of the energy density of the magnetic and electric fields in the entire space and (ii) the dipole contribution −Mm·δH due to the presence of a magnetic particle. The result is

(25) $$\begin{array}{c} \delta U=-\frac{1}{4\pi }\int_{\Omega }\left(H\;\cdot \;\delta H+E\;\cdot \;\delta E\right)dV-\int_{\Omega_{p}}\left(\frac{\varepsilon_{p}-1}{4\pi }+P_{p}\right)\;\cdot \;\delta EdV\\ -\int_{\Omega_{m}}\left(\frac{\varepsilon_{m}-1}{4\pi }\;\cdot \;\delta E+Mm\;\cdot \;\delta H\right)dV+\int_{\Omega_{p}}\left(\sigma_{p}+T_{m}^{\left(e\right)}\right)\;\cdot \;\cdot \delta EdV\\ +\int_{\Omega_{m}}\left(\sigma_{m}+T_{m}^{\left(i\right)}\right)\;\cdot \;\cdot \nabla udV; \end{array}$$

here tensors $\sigma_{p}$ and $\sigma_{m}$ are defined by Formulas (12) and (13), and $T_{m}^{\left(e,i\right)}$ are the magnetic parts of the Maxwell stress tensor; we neglect its electric part. The explicit expressions for the Maxwell stress tensor inside and outside the particle are

(26) $$T_{m}^{\left(i\right)}=\frac{1}{4\pi }\;H⊗\left(H+4\pi Mm\right)-\frac{1}{8\pi }\;H^{2}I,\;T_{m}^{\left(e\right)}=\frac{1}{4\pi }\;H⊗H-\frac{1}{8\pi }\;H^{2}I.$$

Note also that in Formula (26), it is assumed for simplicity that the dielectric permeabilities of the matrix and particles are scalar quantities.

## 4. Numerical Calculation

In the considered quasi-static problem, the magnetic and electric fields are not coupled, so it is convenient to present them in terms of the respective scalar potentials:

(27) $$H=H_{0}-\nabla \psi ,\;E=-\nabla \varphi .$$

The considered ME film is a single-layer system composed of identical elements and infinite in ±Ox and ±Oy directions. Each cell is a cube with dimensions 3ℓ×3ℓ×3ℓ whose lower face is fixed on the z=0 plane. Periodic conditions are imposed on all thermodynamic functions determining the state of the cell along Ox and Oy axes. In the ±Oz directions, the computational domain has a finite size, which, for the correct calculation of the magnetic and electric fields, must significantly exceed the size of the cube. For that, to each cell the space regions in the form of straight prisms with bases 3ℓ×3ℓ and height $h_{\mathrm{sp}}\gg 3\ell$ are adjoined from both sides along the Oz axis, i.e., the actual RVE portion of the film participating in the modeling is a parallelepiped with dimensions $3\ell \times 3\ell \times (3\ell +2h_{\mathrm{sp}})$.

Moreover, to find the elastic stresses in the film, it is necessary that the displacement field u be defined in the entire computational domain. To comply with this requirement, regions $\Omega_{s}=\Omega /\left[\Omega_{p}\cup \Omega_{m,\beta }\right]$ surrounding the film are assigned linear-elastic properties, similar to those in relations (12) or (13). However, the value of modulus $G_{s}$ of this virtual substance is chosen to be several orders of magnitude smaller than the moduli $G_{m}$ and $G_{p}$. Given that, the particular value of $G_{s}$ does not affect the simulation results.

For this reason, when calculating the state of the composite film, we solved not the equation δU=0 from (25) but used the modified expression

(28) $$\delta U^{\prime }=\delta U+\int_{\Omega_{s}}\left[2G_{s}e+\frac{G_{s}\nu_{s}}{1-2\nu_{s}}\mathrm{Tr}(e)I+T_{m}^{\left(e\right)}\right]=0,$$

thereby including the entire computational domain in the consideration.

**Figure 2:**
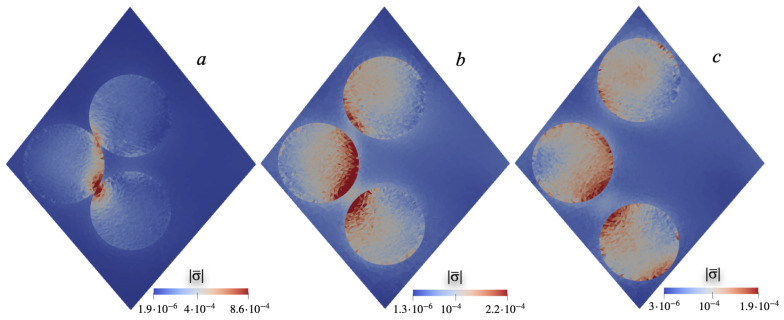
Heat maps of the distribution of stress intensity $|\bar{\sigma }|$ on the diagonal plane of the cell (see Figure 1); interparticle distances: $\bar{l}=$1.63 (**a**), 2.2 (**b**), 2.78 (**c**); applied field ${\bar{H}}_{0}=0.01$; orientation of the piezoelectric anisotropy axis $\vartheta \approx 38^{\circ }$. To avoid confusion, here the overlines denoting dimensionless quantities are made explicit.

**Figure 3:**
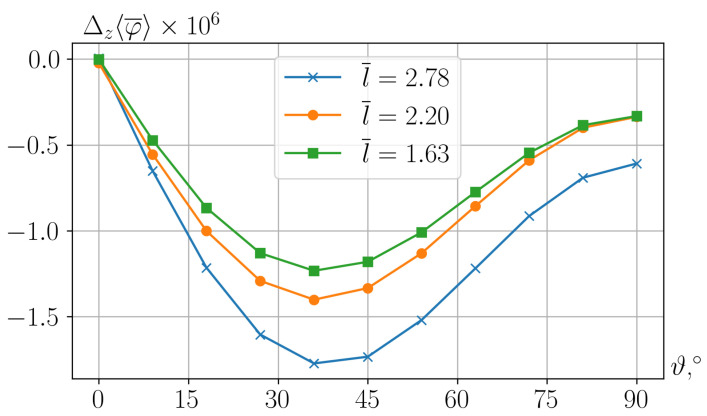
Dependence of the potential difference induced by the field ${\bar{H}}_{0}=0.01$ in cells with different mutual arrangements of particles—see Figure 1—on the angle of orientation of the piezoelectric anisotropy axis. To avoid confusion, here the overlines denoting dimensionless quantities are made explicit.

**Figure 4:**
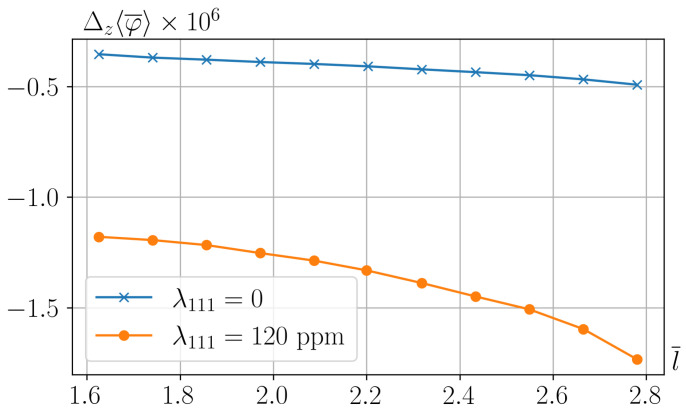
Electric potential difference, induced by the field ${\bar{H}}_{0}=0.01$ in the cell with the optimal orientation of the piezoelectric axis, as a function of the distance $\bar{l}$ between the particles along the line connecting points A and C, see the diagram in Figure 1. For comparison, the same dependence is shown, calculated under the assumption that the ferrite has zero magnetostriction, i.e., all the piezopolarization is due solely to the magnetorotational mechanism. To avoid confusion, here the overlines denoting dimensionless quantities are made explicit.

Before commencing the numerical procedure, Equation (28) was transformed into dimensionless form. For the scaling factor, the value $g=10^{-5}$ with the dimension of inverse square root of the volume energy density was taken: $\left[g\right]=1⁄\left[E\right]=1⁄\left[H\right]=1⁄\left[\sqrt{G_{p,m,s}}\right]$; the same dimension has the components of the piezoelectric tensor. In this representation

(29) $${\bar{d}}_{ik}=d_{ik}/g,\;\bar{H}=gH,\;\bar{E}=gE,\;\Delta \bar{\varphi }=\left(g/\ell \right)\Delta \varphi ,\ldots ;$$

whereas the distances are scaled in the units of length *ℓ*. With this choice, all coefficients in the variational Equation (28) and the algebraic equations defining it fall inside the range 0.1÷10, which significantly facilitates the numerical calculations. Below, in Figure 2, Figure 3 and Figure 4, all the results are given in the aforementioned dimensionless units, but the overline is omitted.

The material and external parameters at which the calculation was performed correspond to a thin film of PVDF@CFO composite, namely

for CFO:

– magnetization $M_{s}=400$ G, anisotropy field $H_{A}=4$ kOe,
– magnetostriction constants $\lambda_{100}=-220$ ppm and $\lambda_{111}=120$ ppm,
– dielectric permittivity $\varepsilon_{m}=100$,
– Young’s modulus $E_{m}=3G_{m}=50$ GPa, Poisson’s ratio $\nu_{m}=0.35$;

for PVDF:

– dielectric permittivity $\varepsilon_{p}=10$,
– Young’s modulus $E_{p}=3G_{p}=2$ GPa, Poisson’s ratio $\nu_{m}=0.3$;
– piezoelectric coefficients [12,33,34,35]: $d_{33}\approx -1.0\times 10^{-6}$, $d_{31}\approx 0.9\times 10^{-6}$,
  $d_{15}\approx -0.7\times 10^{-6}$, all – in CGS units;

for the RVE cell:

– particle radius R=15 nm, cube side ℓ=22 nm,
– applied magnetic field $H_{0}=1$ kOe.

The boundary conditions imposed on the problem variables are

(30) $$u{|}_{z=0}=0,\;\frac{\partial \psi }{\partial z}{|}_{z=-h_{\mathrm{sp}},\;3\ell +h_{\mathrm{sp}}}=0,\;\frac{\partial \varphi }{\partial z}{|}_{z=-h_{\mathrm{sp}},\;3\ell +h_{\mathrm{sp}}}=0$$

meaning that the lower surface of the film is fixed on a solid substrate, and the derivatives of the potentials vanish at the boundaries of the computational domain.

To find the minimum of the energy functional $U^{\prime }$ with respect to unknown functions u,ψ,φ with boundary conditions (30), the finite element method was applied; specifically, its implementation from the FEniCS package version 0.9 for the python language was used. FEniCS [36] is a well-known open-source computational platform for solving partial differential equations.

## 5. Results

The calculation was performed for the RVE cell schematically shown in Figure 1. The easy magnetization axes of all particles are oriented in the film plane in one direction: Ox. External magnetic field $H_{0}$ is directed perpendicular to the axes n of the particles so that the field simultaneously induces both magnetostrictive deformation of the particle and—through the interaction of magnetic moment μ with anisotropy axis n—its rotation, which, of course, is opposed by the elastic resistance of the matrix. The resulting strain fields generate internal stresses in the PVDF matrix, under the influence of which a distributed polarization P and proportional to its internal electric field E arise. Due to the linearity of the piezoelectric problem, the potential difference between the sides of the film (along the Oz axis)—which is a macroscopically measurable quantity—can be found by averaging the corresponding component of the field E over the cell volume and consequent multiplication by the cube side.

The result of numerical calculation is the internal (mesoscale) distributions of mechanical and electric fields in the cell. They ultimately determine the macroscopically measurable result: the potential difference $\Delta_{z}\langle \varphi \rangle$ between the sides of the film. As an example, Figure 2 shows the distributions of the intensity of mechanical stresses $\left|\sigma \right|=\sqrt{\Sigma \sigma_{ik}^{2}}$ on the diagonal plane of the cell at various interparticle distances *l* between particles along the line connecting points A and C, see the diagram in Figure 1; the graphs are plotted for magnetizing field $H_{0}=0.01$.

Please note that when evaluating the internal magnetic field acting on the particle, our calculation fully takes into account both the fields of the particle’s neighbors in the cell and the demagnetizing fields inherent in the film as a whole.

In films of the considered structure, see, for example, ref. [23], the resulting potential difference $\Delta_{z}\langle \varphi \rangle$ significantly depends on the orientation ϑ of the piezoelectric anisotropy axis. Figure 3 shows the dependencies $\Delta_{z}\langle \varphi \rangle \left(\vartheta \right)$ for cells with different mutual arrangements of particles.

As can be seen, the greatest effect is obtained with the piezoelectric axis inclined at angle $\vartheta \approx 40^{\circ }$ to the film plane. In fact, for different interparticle distances *l*, the exact calculated values of this optimal angle differ from one another, but the lines in Figure 3 show that these differences are insignificant. It is noteworthy that estimation $\vartheta \approx 38^{\circ }$ of the extremum of $\Delta_{z}\langle \varphi \rangle \left(\vartheta \right)$ found in the numerical calculation agrees well with the prediction obtained from the solution of the single-particle problem: $\vartheta \approx 41^{\circ }$, see Appendix A.

From Figure 3, it also follows that the best polarization is achieved under maximum separation of the particles from each other. The dependence $\Delta_{z}\langle \varphi \rangle \left(l\right)$ at optimal angles of inclination of the piezoelectric axis is shown in Figure 4.

## 6. Discussion

Compared to the solution for a similar model in Ref. [23], the here-presented calculation is based on more realistic assumptions. Two important steps have been taken in this direction. First, the cubic symmetry of the magnetic anisotropy and magnetostriction in CFO particles has been taken into account; in [23], both these interactions were treated in a simplified manner—as uniaxial. While one may find some grounds for justifying the assumption of uniaxial anisotropy (it is encountered in superparamagnetic nanopowders), uniaxial magnetostriction for CFO particles was an unfounded simplification. This is why the magnetostriction constant entering the results of Ref. [23] could not be associated with any experimentally obtained value. Here, the results include a quite definite, taken from the tables, coefficient $\lambda_{111}$.

The second essential improvement of the model is the consideration of a multi-particle RVE cell. The study of such a structure allows one to clarify how, under the magnetic interaction of particles, the change in their short-range order affects the ME effect. This issue did not make sense within the previous model. Meanwhile, see Figure 3. This factor turned out to be quite significant. Certainly, the obtained result refers to quite a limited number of particle arrangement patterns, whose number is virtually infinite. However, it is important in the qualitative aspect—it proves the relevance of that factor since the approach/separation of particles changes the result by tens of percent.

In the developed linear theory, the magnetostrictive contribution is directly proportional to the cubic magnetostriction constant $\lambda_{111}$. When considering separately the contributions of the magnetostrictive and magnetorotational effects to the transverse polarization of the film, i.e., substituting Formulas (A3) into (A6), one finds for the component $P_{z}\left(\vartheta \right)$:

(31) $$P_{z}=\left[2\left(d_{33}-d_{31}-d_{15}\right)\sin\vartheta \cos^{2}\vartheta +d_{15}\sin\vartheta \right]\;\cdot \;\left(3G_{p}\frac{\lambda_{111}}{H_{A}}+\frac{1}{2}M_{s}\right)H.$$

As can be seen, in the considered problem—i.e., assuming that the polymer matrix is PVDF—the joint action of magnetic striction and magneto-induced rotation is determined by the sign of the parameter $\lambda_{111}$. For positive $\lambda_{111}$, which is the case for iron and cobalt ferrite-spinels, Formula (31) predicts the addition of these contributions; however, for nickel ferrites, for example, this parameter is negative [28]. Note also that formulas alike (31) allow one as well to draw estimations for other combinations of the ME composite components with different $d_{ij}$ and $\lambda_{111}$.

Passing from dimensionless units to dimensional ones, let us estimate the magnitude of the effects that follow from our calculations. For instance, the dimensional potential difference is expressed as $\Delta_{z}\varphi =\ell /g\Delta_{\bar{z}}\langle \bar{\varphi }\rangle$. Let us set, as above, that R=15 nm, i.e., ℓ=R⁄0.69=22 nm. Then the reference dimensionless potential difference $\Delta_{\bar{z}}\langle \bar{\varphi }\rangle ∼1.5\times 10^{-6}$, see Figure 3, obtained in the calculation, transforms into

(32) $$\Delta_{z}\varphi =\ell /g\Delta_{z}\langle \bar{\varphi }\rangle ∼\left(22\times 10^{-7}/10^{-5}\right)\times 1.5\times 10^{-6}\times 300\approx 10^{-4}=100\;\mathrm{mV};$$

the last factor in (32) converts the units of potential difference from CGS to volts. Using this value to determine the magnetoelectric susceptibility of the considered film, one finds

(33) $$\alpha_{V}=\Delta_{z}\varphi /\left(3\ell \;\cdot \;H_{0}\right)\approx 30\;\mathrm{mV}/\left(\mathrm{cm}\;\cdot \;\mathrm{Oe}\right).$$

From the general expressions, see (1) and (2), it follows that the magnetostrictive properties of CFO, like for any crystal of cubic symmetry, are characterized by two significantly different constants. Specifically, whereas $\lambda_{111}∼+120$ ppm, the second parameter has the greatest value among all ferrites: $\lambda_{100}\approx -600$ ppm [28]. The situation where the full set of constants would be involved implies a nonlinear consideration, i.e., the case where the applied field, although not very close to the switching value $H_{A}$, falls beyond the $H/H_{A}\ll 1$ approximation. For $m=(m_{x},0,m_{z})$, retaining the terms quadratic in $m_{z}$ (but neglecting cubic ones), from expansion (1) after subtracting the spontaneous magnetostriction one obtains

(34) $$\lambda =\frac{3}{2}\lambda_{100}\left(q_{z}^{2}-q_{x}^{2}\right)m_{z}^{2}+3\lambda_{111}m_{z}q_{x}q_{z}.$$

Therefore, the quadratic in $m_{z}$ (and, thus in *H*) term prevails only when

(35) $$|\lambda_{100}|\left(H/H_{A}\right)>2\lambda_{111}\;\Rightarrow \;H>2(\lambda_{111}/|\lambda_{100}\left|\right)H_{A}.$$

For the above-given numbers, Equation (35) yields $H>0.4H_{A}$ that for the reference for CFO value $H_{A}∼5$ kOe establishes that $\lambda_{100}$ contribution becomes dominant in the H≳2 kOe range. This demonstrates that, although the $\lambda_{100}$-related contribution becomes important under strong fields, for the here-considered range (up to H∼1 kOe), the linear striction term is fully sufficient.

## 7. Conclusions

Obviously, our calculation is of a model nature, and therefore, only order-of-magnitude agreement with the obtained values should be expected. Even with this limitation, we see that the result (33) in terms of both the used values of static magnetic field strength and attainable potential difference are in fairly good agreement with those required for biomedical experiments with PVDF@CFO films [37]. Indeed, to obtain, for example, a surface potential of ∼50 mV, suitable for successful stimulation of osteogenesis by a field ∼2 kOe [38], according to (33), it is necessary to have a film with thickness of ∼17μm.

The results presented, although being quite positive, of course, are, on the other hand, exhaustive. All the conclusions should be compared with experimental data, which is a task for the future. The main goal of the above-presented study is to show the power of computer modeling in revealing the interconnections between the structure of magnetoelectric composites and the type and magnitude of the expected effects.

We surmise that the approach commenced in Ref. [23] and advanced here—mesoscopic mathematical modeling of ME composites—when appropriately developed, offers the most adequate ‘tool’ to address the nonlinear behavior of such materials. The latter work is a worthy, timely, and quite feasible challenge since detailed modeling of intrinsic interactions in polymer composite multiferroics is yet an almost untouched area of physical materials science.

## Figures and Tables

**Figure A1:**
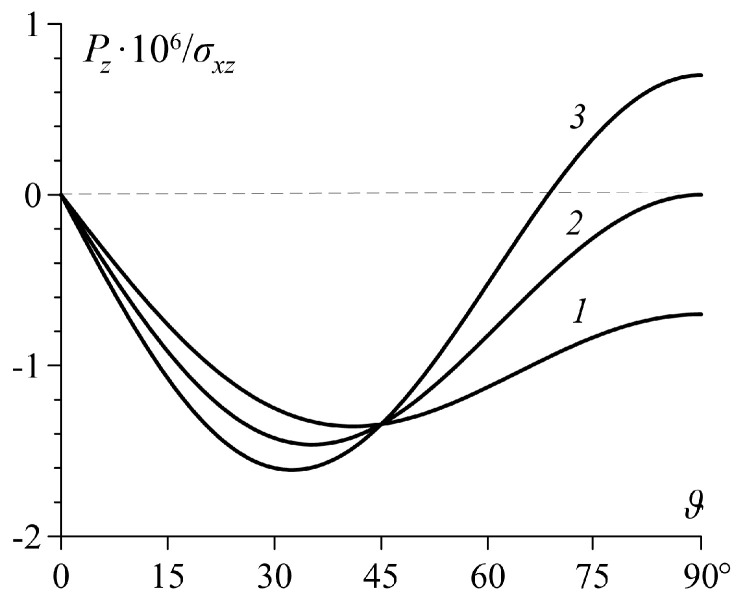
Angular dependences—according to Formula (A6)—of the $P_{z}$ component for the values of coefficient $d_{15}$: −0.7 (*1*), 0 (*2*), 0.7 (*3*); the remaining coefficients: $d_{33}=-1$, $d_{31}=0.9$; all these values are expressed in CGS units with the coefficient $10^{6}$, see Formula (A7).

## Data Availability

Data will be made available upon request.

## Appendix A

As indicated in Section 3, see Formulas (16) and (22), according to the classical consideration [31], the linear piezoelectric effect—the electric polarization arising in a substance under the application of mechanical stress σ—is given by the relation:

(A1) $$P_{i}=\gamma_{i,kl}\sigma_{kl},\;$$

where the tensor γ is symmetric in the last pair of indices. A PVDF sample subjected to poling in the direction ν, in the case of achieving a high degree of orientation, can be considered to be a single piezoelectric domain. In this case, the piezotensor can be represented in the form (21):

(A2) $$\gamma_{i,kl}=A\nu_{i}\delta_{kl}+B\nu_{i}\nu_{k}\nu_{l}+\left(1/2\right)C\left(\nu_{l}\delta_{ik}+\nu_{k}\delta_{il}\right),$$

where A,B,C are material parameters expressed through the piezoelectric coefficients $d_{ik}$, see relations (24).

Consider the case of shear stress, which results from the torque applied to the particle, where the equilibrium magnetization, and hence the axis of magnetic anisotropy of the particles, are directed along Ox, and the external field is along Oz. As it should be, in this case, the stress tensor is symmetric and has a zero trace. This problem corresponds to the stress tensors (8) and (10); each is described by a single parameter $\sigma_{xz}=\sigma_{zx}$:

(A3) $$\sigma_{xz}^{\left(m.\mathrm{strict}\right)}=3G_{m}\lambda_{111}\frac{H}{H_{A}},\;\sigma_{xz}^{\left(m.\mathrm{rot}\right)}=\frac{1}{2}M_{s}H$$

both contributions are linear in the applied field strength H=(0,0,H).

Vector ν is located in the xOz plane:

(A4) $$\nu =\left(\nu_{x},0,\nu_{z}\right)=\left(\sin\vartheta ,0,\cos\vartheta \right).$$

Substitution of (A2) and (A4) into (A1) gives, with account for (A3):

(A5) $$\begin{array}{c} P_{x}\;=\;2\left(d_{33}\;-\;d_{31}\;-\;d_{15}\right)\;\nu_{x}^{2}\nu_{z}\sigma_{xz}\;+d_{15}\nu_{z},\\ P_{z}\;=\;2\left(d_{33}\;-\;d_{31}\;-\;d_{15}\right)\;\nu_{x}\nu_{z}^{2}\sigma_{xz}\;+d_{15}\nu_{x}. \end{array}$$

here component $P_{y}$ is omitted since it is identical to zero. In terms of the angle ϑ, one has

(A6) $$\begin{array}{c} P_{x}\;=\;2\left(d_{33}\;-\;d_{31}\;-\;d_{15}\right)\;\sin^{2}\vartheta \cos\vartheta \sigma_{xz}\;+d_{15}\cos\vartheta ,\\ P_{z}\;=\;2\left(d_{33}\;-\;d_{31}\;-\;d_{15}\right)\;\sin\vartheta \cos^{2}\vartheta \sigma_{xz}\;+d_{15}\sin\vartheta . \end{array}$$

Substituting for estimations the values of $d_{ik}$ indicated in Section 4, one finds

(A7) $$\begin{array}{c} P_{x}\;\cdot \;10^{6}/\sigma_{xz}=-2.4\sin^{2}\vartheta \cos\vartheta -0.7\cos\vartheta ,\\ P_{z}\;\cdot \;10^{6}/\sigma_{xz}=-2.4\sin\vartheta \cos^{2}\vartheta -0.7\sin\vartheta . \end{array}$$

Then, the extrema of the polarization components (A7) as functions of the angle are easily found. Namely (with the coefficient $10^{6}$ and at $\sigma_{xz}=1$) they are:

(A8) $$\begin{array}{ccc} P_{x}\left(0^{\circ }\right)=-0.7, & \;\;P_{x}\left(90^{\circ }\right)=0, & \;\;P_{x}\left(49^{\circ }\right)=-1.36;\\ P_{z}\left(0^{\circ }\right)=0, & \;\;P_{z}\left(90^{\circ }\right)=-0.7, & \;\;P_{z}\left(41^{\circ }\right)=-1.36. \end{array}$$

As can be seen from (A8), the maximal polarizations are achieved in configurations where the piezoelectric anisotropy axis is oriented at a significant angle to the film plane. The explanation follows from Formulas (A6) and (A7): when the poling direction is around $45^{\circ }$, not only the single ‘tangential’ component $d_{15}$ of the piezotensor (as at angles 0 and $90^{\circ }$) contributes to the polarization, but its ‘diagonal’ components also have a significant influence. Moreover, for different components of vector P, the orientations ν which provide the maximum, do differ.

We remark that in the literature on the properties of PVDF, the main interest has always been focused on clarifying the values of the ‘diagonal’ components. Their values are fairly well established, the results of numerous measurements do not differ too much [12,33,34,37,39,40,41,42]. The values of $d_{33}$ and $d_{31}$ taken for the estimates in Section 4 correspond to known experimental results. At the same time, there are significant discrepancies in the literature regarding coefficient $d_{15}$, both in absolute value $\left(0.2÷1.1\right)\times 10^{-6}$ and in sign. In most measurements, parameter $d_{15}$ in PVDF is indicated as negative [34,37,40], but there are works where positive values of $d_{15}$ are reported, see [42], for example.

In Section 4, the value $d_{15}=-0.7\times 10^{-6}$ [CGS units] was taken as an estimation and further used in numerical calculations. However, Formula (A8) allows one to trace how the magnitude of this coefficient affects the angular dependence of the polarization directed across the film plane. As shown, the set of reference values of $d_{ik}$ adopted in Section 4, the dependence $P_{z}\left(\vartheta \right)$ has a minimum near $\vartheta \approx 41^{\circ }$. Figure A1 illustrates the changes occurring when coefficient $d_{15}$ is varied; the curves $P_{z}\left(\vartheta \right)$ shown there correspond to negative, zero, and positive values of $d_{15}$.

Notably, this purely model single-particle problem yields function $P_{z}\left(\vartheta \right)$ that is qualitatively close to those obtained from the solution of the full RVE problem: a film of many cells and several particles in each of them. For that it suffices to compare curve *1* in Figure A1 and the family of lines in Figure 3, all obtained at $d_{15}=-0.7\times 10^{-6}$. This similarity suggests that the strong dependence on $d_{15}$ predicted by Figure A1: (i) the maximum of $P_{z}\left(\vartheta \right)$ at the end of the angular interval and (ii) the position of its internal extremum, would most likely be inherent in the results of the RVE problem as well. Certainly, it might be only a qualitative similarity. The simple calculation performed above is not able to explain, for example, the dependence of function $P_{z}\left(\vartheta \right)$ on the spatial distribution of particles inside the cell.

As a special feature, we note that with a suitable sign and magnitude of $d_{15}$, the sign of the transverse polarization, and hence the sign of the induced potential difference on the film, can undergo inversion; the zero level $\Delta_{z}\varphi$ is indicated by the dashed line in Figure A1.

In other words, a film with the same internal arrangement (structure) of the composite, depending on the poling direction, can create a potential of either sign in response to the same magnetic field.
